# An assessment of O_3_-related health risks and economic losses in typical regions of China

**DOI:** 10.3389/fpubh.2023.1194340

**Published:** 2023-09-05

**Authors:** Xiaowei Song, Yongpei Hao

**Affiliations:** College of Resources and Environment, Shanxi University of Finance and Economics, Taiyuan, China

**Keywords:** O_3_, distribution characteristic, meteorological factors, health effect, economic loss, Fenwei plain

## Abstract

**Introduction:**

As one of the key areas for air pollution prevention and control in China, the Fenwei Plain is experiencing serious near-surface O_3_ pollution, which is a key issue that needs to be solved urgently.

**Methods:**

Based on pollutant concentration monitoring data and meteorological and health data over the same period, this study analyzed the temporal and spatial characteristics, the relationships with meteorological factors of O_3_ pollution, and the health effects and economic losses caused by exposure to O_3_ pollution using environmental health risk and environmental value assessment methods in 11 cities on the Fenwei Plain in China from 2014 to 2020.

**Results:**

The results showed that O_3_ pollution has become increasingly serious on the Fenwei Plain in recent years. The annual average concentration of O_3__8h_max showed an overall upwards trend, with an increase of 32.39% in 2020 compared to 2014. The mean concentrations observed in summer were the highest, followed by spring and autumn, and the lowest was in winter. The O_3_ concentration had a significant positive correlation with air temperature and sunshine hours. The evaluation results of the impact of air pollution on population health showed that the number of premature deaths caused by O_3_ pollution fluctuated and increased during 2014–2020. In 2020, the numbers of total, cardiovascular and respiratory deaths attributable to O_3_ pollution on the Fenwei Plain were 6,867 (95% CI: 3,739–9,965), 3,652 (95% CI: 1,363–5,905), and 1,257 (95% CI: 747–2,365), respectively, and the total number of premature deaths related to O_3_ exposure increased by 48.05% compared with 2014. The health and economic losses attributed to O_3_ pollution on the Fenwei Plain during the study period were 44.22 (95% CI: 22.17–69.18), 47.16 (95% CI: 23.64–73.77), 68.28 (95% CI: 34.27–106.31), 114.44 (95% CI: 57.42–177.76), 110.85 (95% CI: 55.45–172.52), 116.41 (95% CI: 58.24–180.74), and 116.81 (95% CI: 58.00–180.88) billion yuan, respectively. In Linfen City, the increasing rate of the number of premature deaths reached 283.39% because the O_3_ concentration increased greatly.

**Discussion:**

Due to high O_3_ concentrations and obvious population growth in Xi’an, the problems of premature death and health and economic losses attributed to O_3_ concentrations exceeding the standard value are prominent.

## 1. Introduction

Ozone (O_3_) in the troposphere is mainly generated by nitrogen oxides (NO_X_) and volatile organic compounds (VOCs) through a series of complex photochemical reactions and is an important pollutant in the atmosphere that affects the air quality of regions and cities (1, 2). In recent years, O_3_ pollution in China has become increasingly severe (3). From 2015 to 2020, the O_3_ concentration and the proportion of days exceeding the standard in 338 cities in China increased each year, while the proportion of cities reaching the standard decreased each year, and the O_3_ concentration in some cities exceeded the second-level concentration limit of the national standard (160 μg/m^3^). For example, O_3_ has become the major pollutant next to PM_2.5_ in the Beijing-Tianjin-Hebei region. O_3_ has replaced PM_2.5_ as the primary annual pollutant in some years in the Pearl River Delta region and the Yangtze River Delta region. O_3_ and PM_2.5_ have become the main pollutants in the atmospheric environment in China. How to curb O_3_ emissions while controlling PM_2.5_ pollution is an urgent issue for air pollution prevention and control, which should be given more attention by the government and the public.

Studies on the spatiotemporal variation in O_3_ pollution have shown that the regional problems of O_3_ pollution in China are significant (4–6). Yi et al. (7) analyzed the variation characteristics of O_3_ concentration in 25 cities in the Yangtze River Delta region and concluded that the spatial distribution of O_3_ pollution shows an obvious patchy distribution with the most serious pollution in Shanghai and its surrounding cities, and meteorological factors such as sunshine duration, air temperature, wind speed and direction, and relative humidity have significant impacts on O_3_ pollution. Hu et al. (8) analyzed the spatiotemporal variation characteristics of O_3_ concentration in the Beijing-Tianjin-Hebei region and concluded that the mass concentration of O_3_ increased yearly from 2014 to 2018, the concentration growth rate of O_3_ in different regions was significantly different, and the O_3_ high value belt was concentrated in Tianjin, Qinhuangdao, and Tangshan. Sun et al. (9) showed that the O_3_ concentration in suburban areas of Guangzhou was lowest in spring and highest in summer, the diurnal variation trend was unimodal, and the O_3_ concentration showed a significant negative correlation with the NO_2_ and CO concentrations. In addition, the O_3_ concentration is significantly affected by the emission of precursor pollutants, and the influence of meteorological conditions on the O_3_ concentration cannot be ignored. Relevant studies have shown that temperature, relative humidity, sunshine duration, and wind speed are significantly correlated with O_3_ concentration, and the seasonal variation characteristics are obvious (10).

The increase in O_3_ concentration near the ground can cause serious harm to human health, construction, agriculture, and animal husbandry (11–14). Exposure to high levels of O_3_ can cause respiratory and cardiovascular disease, and prolonged exposure to relatively low concentrations also increases the risk of premature death (15, 16). Studies on several southern cities in China have shown that O_3_ has the greatest impact on respiratory mortality (17). Current studies on O_3_ have mainly focused on short-term exposure and acute effects, and the results show that O_3_ is significantly correlated with a variety of diseases, especially cardiovascular diseases, showing independent health hazards, spatial differences, and spatial spillover effects and is sensitive to season. At present, there have been many quantitative studies on the health effects caused by O_3_ pollution, among which the integrated exposure-response (IER) model is mainly used for evaluation. For example, Liu et al. (18) used data from 1,516 monitoring stations in China combined with the air quality model and found that the excess deaths of COPD (chronic obstructive pulmonary disease) related to O_3_ concentration exposure in China in 2015 were approximately 55,341–80,280 people. Zeng et al. (19) found that the number of premature deaths caused by O_3_ pollution in China in 2017 was 9.8 × 10^4^. Feng et al. (20) estimated that the economic loss caused by O_3_ pollution to the health risk of Chinese residents in 2015 was as high as US $698.36 billion, accounting for approximately 6.3% of the GDP at that time.

At present, studies on the health effects of O_3_ pollution have mainly focused on epidemiological studies, which have been concentrated in economically developed cities and regions such as the Yangtze River Delta, Pearl River Delta, and Beijing-Tianjin-Hebei (17, 21–26), and there has been a lack of studies on the spatiotemporal distribution and health effect assessment of O_3_ pollution in economically underdeveloped areas. Similar to developed areas, atmospheric pollutant emissions in the central and western regions under the background of rapid urbanization have increased significantly, especially in regard to industrial transfers to developed areas at the same time, causing these areas to be inevitably faced with the choice of economic development and environmental protection or serious atmospheric pollution problems such as O_3_, endangering population health. As an important urban agglomeration and energy supply area in central and western China, the Fenwei Plain has become a key area for air pollution prevention and control in China, with O_3_ pollution in the region becoming increasingly serious. From 2017 to 2019, O_3_ pollution on the Fenwei Plain became increasingly serious, and the annual average concentration of the 90th percentile of the daily 8 h maximum reached 184 μg/m^3^. At the same time, different cities on the Fenwei Plain are located in different landforms, and their sources and variation characteristics of pollutants are also different. It is urgent to study the characteristics of O_3_ pollution in this region, the relationship between O_3_ pollution and meteorological factors, and the damage to human health. In addition, there are significant differences in air pollution and population distribution in different cities on the Fenwei Plain, which requires accurate coupling of pollutant concentration and population exposure data at the spatial scale to refine and assess the impact of air pollutants on population health in different cities in the region.

Therefore, based on continuous monitoring data of the O_3__8h_max concentration near the surface in 11 cities on the Fenwei Plain from 2014 to 2020 and meteorological data during the same period, this study analyzed the spatial and temporal variation characteristics of the O_3__8h_max concentration from different scales and analyzed the relationship between meteorological factors and O_3_ concentration variation. On this basis, with the secondary concentration limit of an ambient air quality standard (GB3095-2012) of 70 μg/m^3^ as the benchmark concentration, this study also evaluated the health effects and health economic losses attributable to O_3_ and compared the evaluation results of different cities and different regions to provide a scientific basis for the health benefits of the implementation of air quality standards in the study area. This study provides a reference for air quality management, health warning and prevention in the study area.

## 2. Methods

### 2.1. Data sources

**Figure 1:**
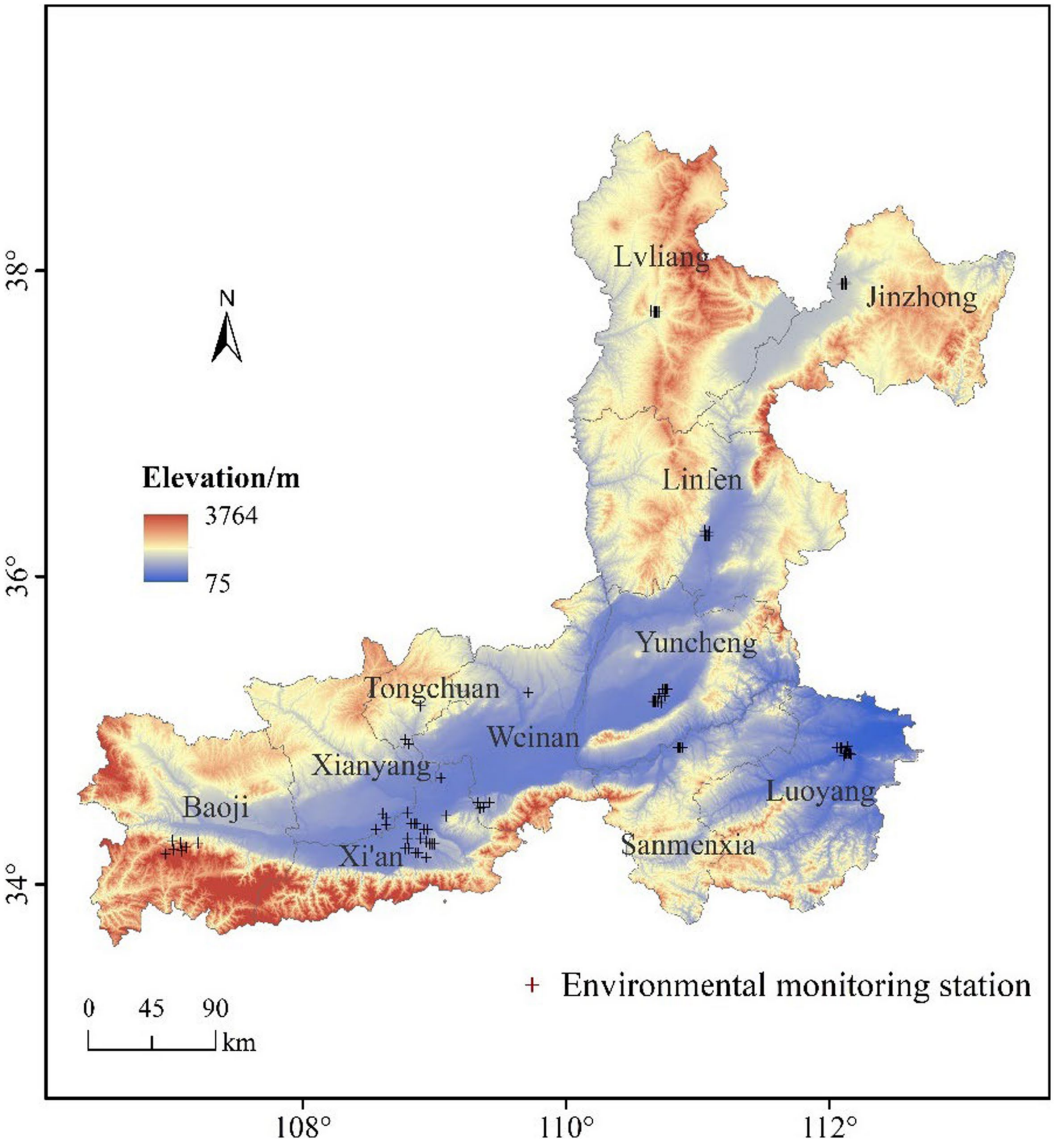
The study region and the distributions of monitoring stations.

The Fenwei Plain (33.33–34.42°N, 106.20–114.07°E) is located in central China and includes the cities of Xi’an, Xianyang, Baoji, Tongchuan, and Weinan in Shaanxi Province; Lvliang, Jinzhong, Linfen, and Yuncheng in Shanxi Province; and Luoyang and Sanmenxia in Henan Province (Figure 1). The Fenwei Plain is composed of Fenhe Plain, Weihe Plain, and their terraces, and is the largest impact plain in the middle reaches of the Yellow River. Among them, the landforms of Lvliang, Jinzhong, and Tongchuan are mainly mountainous and fluctuated greatly; Sanmenxia is located in the valley topography of the terrace along the Yellow River, and its elevation fluctuates obviously; Luoyan is located in a relatively flat terrain, the northeast plain area is relatively large; The plain area of Yuncheng accounts for 60.6% of the total area, and the terrain types are plains, hills, and low mountains; Linfen is surrounded by mountains, the pollutants are not easy to spread; While Weinan, Xi‘an, Xianyang, and Baoji are located in the lower reaches of the Wei River valley alluvial plain, surrounded by mountains on three sides, high in the west and low in the east. The Fenwei Plain is a warm temperate semi-humid climate with an average annual temperature of 6–14°C and annual precipitation of 420–900 mm, mainly from May to September.

The atmospheric O_3_ concentration data from 11 cities on the Fenwei Plain from 2014 to 2020 were obtained from the Environmental Monitoring Station of the Ministry of Ecology and Environment of China. Meteorological data from the same period (sunshine duration, temperature, relative humidity, wind direction, wind speed, and precipitation data) were derived from daily mean and hourly observation data from meteorological stations in 11 cities. Due to the lack of data, the pollutant concentration data from Jinzhong, Lvliang, and Yuncheng were collected from 2015 to 2020, while data from the other eight cities were collected from 2014 to 2020. The annual mean value of the mass concentration of O_3__8h_max pollutants (moving average of the maximum 8-h concentration) refers to the arithmetic mean value of O_3__8h_max on all days in the study year, the quarterly mean value refers to the arithmetic mean value of O_3__8h_max on all days in the research season, the monthly mean value refers to the arithmetic mean value of O_3__8h_max on all days in the research month, and the daily mean value refers to the moving average value of the daily maximum 8-h concentrations. In this paper, the daily O_3__8h_max was selected as the exposure index of the O_3_ concentration. The average annual O_3_ concentration used was the mean mass concentration of the 90th percentile of O_3__8h_max to evaluate the health effects of exposure. The daily O_3_ concentration exceeding the standard value indicates that the O_3__8h_max concentration is higher than 160 μg/m^3^. An hourly O_3_ concentration exceeding the standard value indicates that the hourly O_3_ concentration is higher than 200 μg/m^3^.

The permanent resident populations of the 11 cities on the Fenwei Plain from 2014 to 2020 are shown in Table 1.

**Table 1:** The population of each city on the Fenwei Plain.

Population/10^4^	2014	2015	2016	2017	2018	2019	2020
Xi’an	862.75	870.56	883.21	961.67	1000.37	1020.35	1295.29
Xianyang	495.08	497.24	498.66	437.6	436.61	435.62	395.98
Weinan	534.3	535.99	537.16	538.29	532.77	527.81	468.87
Baoji	375.32	376.33	377.5	378.1	377.1	376.1	332.19
Tongchuan	84.51	84.62	84.72	83.34	80.37	78.01	69.83
Lvliang	381.31	383.22	385.48	387.88	388.56	389.08	339.84
Jinzhong	332.03	333.56	334.87	336.55	338.15	338.94	337.95
Linfen	441.45	443.56	445.8	448.15	450.02	450.83	397.65
Yuncheng	525.22	527.53	530.51	533.6	535.96	537.26	477.45
Luoyang	668.26	674.55	680.14	682.55	689.01	692.55	705.67
Sanmenxia	225.14	225.1	226.98	227.98	227.25	228.01	203.49

### 2.2. Population health impact assessment

In this study, air pollution affected the population health function and was used to estimate the excess number of deaths related to O_3_ (27). Based on previous studies (28, 29), three health endpoints were considered in this study: total mortality (Total), cardiovascular disease mortality (CVD), and respiratory disease mortality (RD). When the concentration of air pollutants exceeded the highest concentration value *C_0_* without causing health damage to the human body, the relative risk index (RR) of a certain disease was calculated based on the results of epidemiological investigations and the current concentration of air pollutants. The excess number of deaths from a disease related to air pollution is expressed as *∆Mort* and calculated as follows:


(1) $$\Delta \mathrm{Mort}=B_{i}\times \mathrm{Pop}\times \left[1-1/RR_{i}\right]$$

where ΔMort —O_3_ is the number of premature deaths of health end-point *i* due to O_3_ exposure; *B_i_* is the baseline mortality of health end-point *i*; *Pop* is the exposed population; and *RR_i_*—O_3_ is the relative risk of health end-point *i* due to O_3_.

*RR* was calculated by the log-linear model as follows:


(2) $$RR=\exp\left[\beta_{i}\left(C-C_{0}\right)\right]$$

In the formula, *β* is the exposure response factor of health terminal *I*; *C* is the O_3__8h_max concentration; and *C_0_* is the O_3__8h_max concentration threshold, below which the health risk can be negligible (Table 2). *β* is an empirical coefficient obtained from a large number of epidemiological investigations based on different pollutants and excess deaths from related diseases.

**Table 2:** Relevant parameters for health risk assessment models.

Pollutant	Health end-point	RR	β	C_0_(μg/m^3^)	References
O_3_	Total	1.0024 (95% CI:1.0013–1.0035)−10 μg/m^3^	0.00024 (95% CI:0.00013–0.00035)	70 (WHO)	(25) (30)
	CVD	1.0027 (95% CI:1.001–1.0044)−10 μg/m^3^	0.00027 (95% CI:0.0001–0.00044)		
	RD	1.0051 (95% CI:1.0003–1.0098)−10 μg/m^3^	0.00051 (95% CI:0.0003–0.00098)		

With regard to concentration thresholds (*C_0_*), the World Health Organization (WHO) stated that the global natural background concentration of O_3_ is about 70 μg/m^3^ (31). Referring to relevant studies, 70 μg/m^3^ was selected as the concentration threshold in this study (32). The baseline mortality data were obtained from the China Statistical Yearbook (33) and the China Health Statistical Yearbook (34) from 2015 to 2021. Due to the unavailability of baseline mortality data at the regional and city levels, we assumed that baseline mortality rates were evenly distributed in China (26), as shown in Table 3.

**Table 3:** Baseline mortality data of cities on the Fenwei Plain.

*B_i_*(10^−5^)	Total	CVD	RD
2014	639.59	287.55	77.10
2015	642.26	289.55	76.66
2016	614.19	272.87	69.03
2017	647.46	297.22	72.89
2018	659.51	306.4	72.85
2019	627.86	286.31	65.02
2020	634.68	300.37	55.36

### 2.3. Health economic benefits assessment

In this study, the value of the statistical life (VSL) was used to estimate the economic cost of premature deaths from environmental exposures; that is, on the basis of the value of statistical life, a hypothetical market considering the risk of death was established (35). VSL, which denotes economic value, is calculated in investigation studies to assess individuals’ willingness to pay (WTP) to reduce mortality risk and has been widely used in the assessment of health economic losses from air pollution (36–38). VSL is mainly determined by gross domestic product (GDP) and consumer price index (CPI). This study assumed that individual WTP increases with income, while GDP *per capita* varies widely across regions of China, and VSL estimates for specific provinces are lacking. Therefore, this study adopted the benefit conversion method (as follows); that is, after adjusting the *per capita* GDP difference, the VSL of a specific province was derived from the benchmark VSL of the existing province (36, 39).


(3) $$VSL_{k,t}=VSL_{\mathrm{base}}\times {\left(\frac{G_{k,t}}{G_{\mathrm{base}}}\right)}^{\gamma }\times {\left(1+\%\Delta P_{k}+\%\Delta G_{k}\right)}^{\gamma }$$

where $VSL_{k,t}$ represents the adjusted *VSL* value of province *k* in year *t*; $VSL_{\mathrm{base}}$ represents the benchmark *VSL*, and this study selected the *VSL* results of Beijing in 2012, with a value of US$132,000 (approximately 936,000 yuan) (36, 40, 41). $G_{k,t}$ represents the *per capita* GDP of study region *k* in year *t*; $G_{\mathrm{base}}$ represents the *per capita* GDP of the base year, i.e., the *per capita* GDP of Beijing in 2012; and *γ* is the income elasticity coefficient, recommended by the Organization for Economic Cooperation and Development (OECD) as 0.8 (42). $\%\Delta P_{k}$ represents the percentage change in the consumer price index (CPI) for study area *k* from the base year to year *t*. $\%\Delta G_{k}$ represents the percentage change in the *per capita* GDP of study region k from the base year to year *t*.

The number of deaths related to pollution exposure calculated by Equation (1) is multiplied by Equation (3) to obtain the total health economic burden of the study area in a given year, as shown in Equation (4) below:


(4) $$E=VSL_{k,t}\times \Delta \mathrm{Mort}$$

## 3. Results and discussion

### 3.1. Variation characteristics of O_3_ pollution

#### 3.1.1. Annual variation characteristics of O_3_ pollution

**Figure 2:**
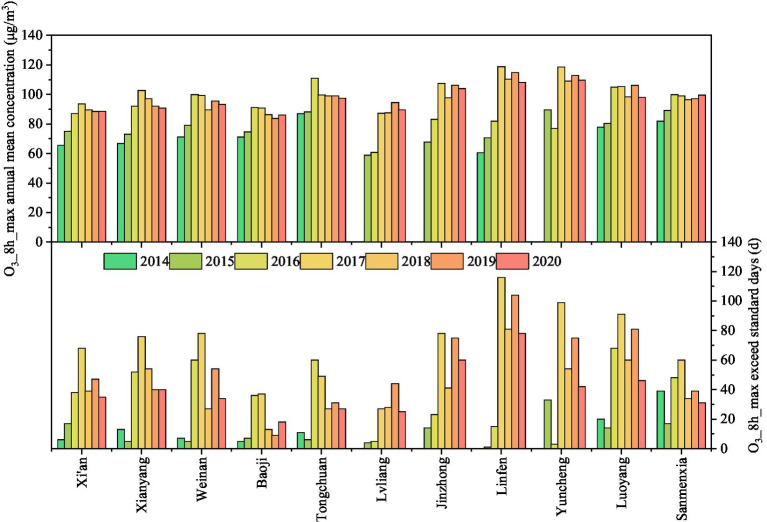
Over-standard days and annual average concentration of O_3__8h_max on the Fenwei Plain from 2014 to 2020.

From 2014 to 2020, the interannual variation in the O_3_ concentration in cities on the Fenwei Plain generally showed an upwards trend (Figure 2). The average annual concentration of O_3__8h_max in each city in 2020 increased by 34.94, 36, 31.06, 21.12, 12.08, 52.48, 53.44, 78.78, 22.43, 26.15, and 21.61% relative to 2014, respectively (data from Lvliang, Jinzhong, and Yuncheng in 2014 were missing and were replaced by data from 2015). Among them, the increase in the O_3__8h_max concentration in Linfen was the most obvious, which was related to the unfavorable diffusion conditions of the flare topography and the high-intensity emission sources. The number of days in which the O_3_ concentration exceeded the standard value generally increased and fluctuated. In particular, the number of days in which the O_3_ concentration exceeded the standard value on the Fenwei Plain reached 70.8 days in 2017, an increase of 33.7 days compared with 2016. The increasing trend of the number of days exceeding the standard value in Linfen City was unusually obvious. Although the O_3_ concentration in cities has shown a decreasing trend in recent years, sudden heavy O_3_ pollution events may also occur. In the future, more efforts should be made to promote O3 pollution emission reduction based on different urban topographic characteristics, especially for Linfen and Yuncheng in the middle of the Fenwei Plain, from various aspects, such as industrial structure, energy utilization, and transportation.

#### 3.1.2. Quarterly variation characteristics of O_3_ pollution

**Figure 3:**
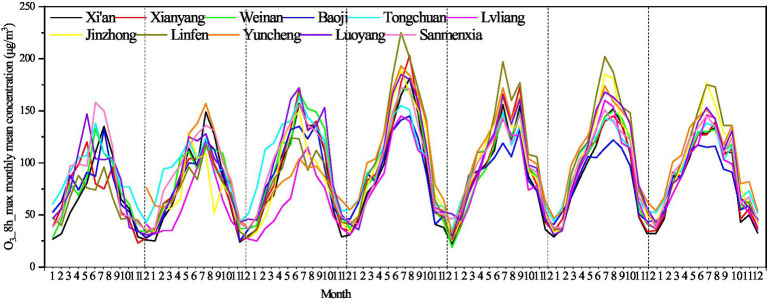
Monthly average concentration of O_3__8h_max on the Fenwei Plain from 2014 to 2020.

The seasonal variation characteristics of the O_3__8h_max concentration in the 11 cities on the Fenwei Plain are shown in Figure 3. The O_3__8h_max concentration was at a low level in winter (December–February), began to show an upward trend in spring (March–May), decreased in June in summer, and continued to rise from July to September, reaching the highest concentration in a year. The order of the O_3__8h_max concentration in the four seasons was ranked as summer >spring >autumn >winter. In summer, with high temperature, strong radiation and strong photochemical reactions, precursors such as NOx and VOCs easily form O_3_ under strong photochemical reactions. In winter, when the weather is more stable, the increase in particle concentration prevents solar radiation, weakens the intensity of solar radiation, limits photochemical reactions, and thus inhibits the generation of O_3_ pollutants (43). The high and rising O_3_ concentration in spring (April to May) is related to the emergence of the upper tropospheric folding effect. Related studies have shown that the tropopause folding effect leads to air exchange between the troposphere and stratosphere, thus increasing the tropospheric O_3_ concentration (44, 45).

Since 2017, the concentration in Linfen in summer (June to August) has been the highest among all cities, with mean concentrations of 199, 179, 181, and 161.33 μg/m^3^, respectively. The concentrations of O_3__8h_max pollutants in Lvliang and Baoji were relatively low in each month.

#### 3.1.3. Diurnal variation characteristics of O_3_ pollution

**Figure 4:**
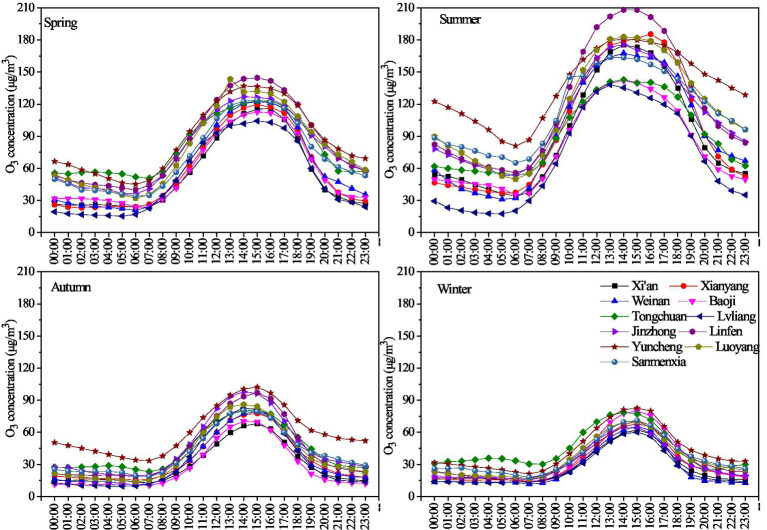
Daily variations in the O_3__8h_max concentrations in the four seasons from 2014 to 2020 on the Fenwei Plain.

The diurnal variations in the average hourly O_3_ concentration in different seasons on the Fenwei Plain during 2014–2020 are shown in Figure 4. The diurnal variation in O_3_ concentration in the 11 cities on the Fenwei Plain showed obvious single-peak and single-valley distributions. The O_3_ concentration was low in the morning and at night, with the lowest value appearing between 06:00 and 08:00. After 08:00, the O_3_ concentration gradually increased, and the high value period was mainly concentrated between 12:00 and 19:00. The appearance of the maximum O_3_ concentration in the afternoon was related to the strong solar radiation and high temperature. The peak time of the O_3_ concentration in summer was earlier than that in the other three seasons and then showed a downward trend. There were slight differences among the 11 cities on the Fenwei Plain in the peak and valley values, the rate of concentration change, and the time of the O3 concentration peak value. The valley value of the O_3_ concentration in the 11 cities in different seasons was very close, and Weinan and Lvliang were the lowest. The peak value of the O_3_ concentration in Yuncheng and Linfen was significantly higher than that in other cities in different seasons, and the change curve of the O_3_ concentration in Yuncheng had the largest slope and amplitude.

### 3.2. The relationship between O_3_ pollution and meteorological conditions

Meteorological conditions are one of the main factors affecting O_3_ concentration. The formation, deposition, transmission, and dissipation of O_3_ are closely related to meteorological conditions. As the O_3_ concentration on the Fenwei Plain in 2017 showed a significant rising trend, which was significantly higher than that in other years, to better analyze the relationship between the change in O_3_ concentration on the Fenwei Plain and meteorological conditions, O_3_ concentration monitoring data in 2017, and meteorological data from the same period were selected for analysis. The relationship between sunshine duration and O_3_ concentration was analyzed using daily data. To more accurately analyze the relationship between different meteorological elements and near-surface O_3_ concentrations, this study used hourly data to analyze the correlation between hourly meteorological observation data (temperature, relative humidity, and wind speed), excluding precipitation periods, and O_3_ concentration data from corresponding periods between 8:00 and 19:00 h in the 11 cities on the Fenwei Plain in 2017.

#### 3.2.1. Sunshine duration

**Figure 5:**
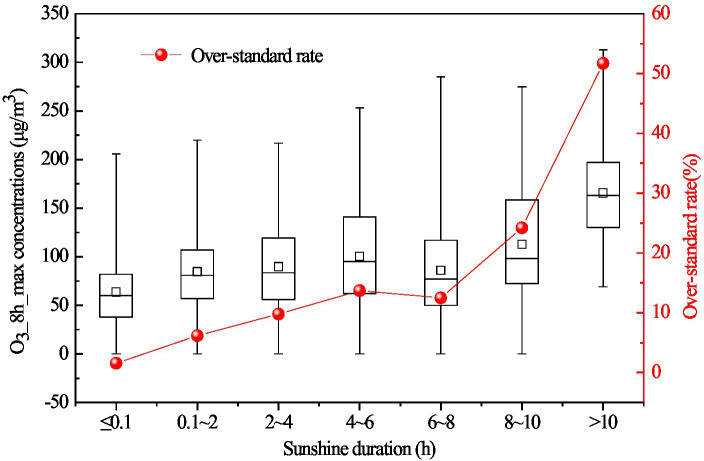
Variations in O_3__8h_max concentration and the over-standard rate with different sunshine hours in 2017 on the Fenwei Plain.

The influence of solar radiation on O_3_ concentration is mainly reflected in the sunshine duration. With the increase of sunshine duration, the photochemical reaction rate increases correspondingly, which accelerates the generation of O_3_. The changes in O_3_ concentration and over-standard rate in different sunshine duration intervals on the Fenwei Plain in 2017 are shown in Figure 5. With the continuous increase of sunshine duration, the O_3__8h_max concentration increases correspondingly, and the over-standard rate of O_3__8h_max concentration also showed an upward trend (Figure 5). The over-standard rate of the O_3__8h_max concentration increases significantly when the sunshine duration exceeds 8 h. Especially, when the sunshine duration is longer than 10 h, the over-standard rate of the O_3__8h_max concentration reached 51.68%, and the average concentration is 165.49 μg/m^3^.

In addition, when the sunshine duration is less than 0.1 h, the O_3__8h_max concentration in very few periods was more than 160 μg/m^3^, and the over-standard rate was 1.56%, which may be related to the vertical and horizontal transport of O_3_. Although sunshine duration has a significant effect on ground-level O_3_ concentrations, the influence of other external conditions on O_3_ concentrations should not be ignored.

#### 3.2.2. Temperature

**Figure 6:**
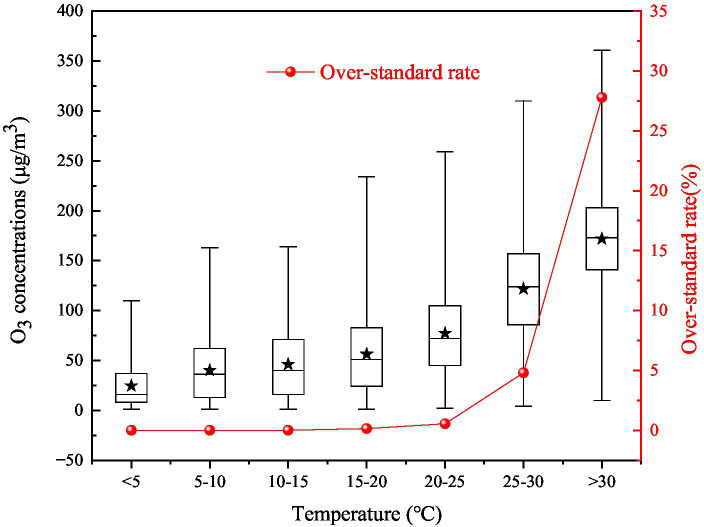
Variations in the O_3_ concentration and the over-standard rate with different temperatures in 2017 on the Fenwei Plain.

Ozone is generated by photochemical reactions under the action of solar radiation, and the intensity of the photochemical reaction is affected by air temperature. The average O_3_ concentration and its over-standard rate have an increasing trend with increasing temperature (Figure 6). When the temperature is lower than 15°C, the O_3_ concentration is less than 49 μg/m^3^ and does not exceed the standard value. And the over-standard rates of O_3_ concentration are 0.14 and 0.54%, respectively, when the temperature ranges are 15–20 and 20–25°C. The over-standard rate of O_3_ concentration increases significantly when the temperature exceeds 25°C. In particular, when the temperature exceeds 30°, the over-standard rate reaches 27.80%, and the average concentration is 171.90 μg/m^3^.

#### 3.2.3. Relative humidity

**Figure 7:**
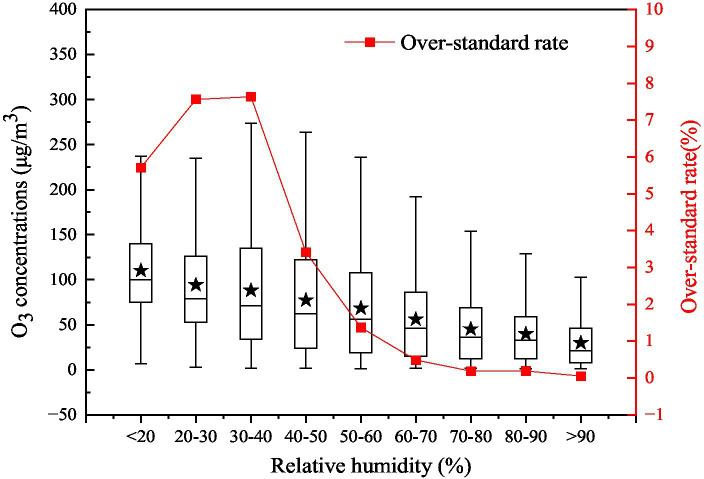
Variations in the O_3__8h_max concentration and the over-standard rate with different relative humidities in 2017 on the Fenwei Plain.

Under certain conditions, water vapor can affect photochemical reaction in the atmosphere, and then affect the concentration of O_3_ in the atmosphere (46). The average O_3_ concentration decreased gradually with the increasement of relative humidity, while the over-standard rate of O_3_ concentration increased first and then decreased with increasing relative humidity (Figure 7). When the relative humidity was lower than 40%, the over-standard rate of O_3_ concentration increased with increasing relative humidity, reaching a maximum of 7.64% and an average mass concentration of 93.95 μg/m^3^. When the relative humidity was greater than 40%, the over-standard rate decreased rapidly with increasing relative humidity, which was related to the fact that the near-surface atmosphere tends to produce wet deposition when the relative humidity is high, which results in the decrease of O_3_ concentration.

#### 3.2.4. Wind speed

**Figure 8:**
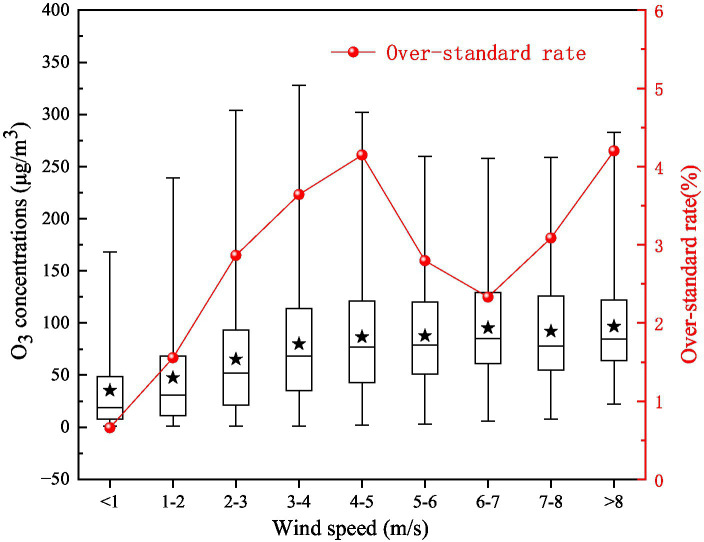
Variations in the O_3__8h_max concentration and the over-standard rate with different wind speeds in 2017 on the Fenwei Plain.

Wind speed has a significant impact on atmospheric stability, and different wind speeds also determine the removal efficiency and transport efficiency of O_3_ concentration. When the wind speed is less than 5 m/s, the over-standard rate of O_3_ concentration have an upward trend with increasing wind speed (Figure 8). When the wind speed is between 4 and 5 m/s, the over-standard rate of O_3_ concentration reach the highest value, 4.15%, and the average mass concentration is 86.59 μg/m^3^. When the wind speed is 7 m·s^−1^, the excess rate of O_3_ concentration show a rising trend again, which may be related to the regional transmission of O_3_. Low wind speed is conducive to photochemical reaction and agglomeration of O_3_ and its precursors, resulting in high concentration of O_3_ pollution. When the wind speed increases gradually, O_3_ and its precursors may be transported across regions, resulting in regional pollution.

**Figure 9:**
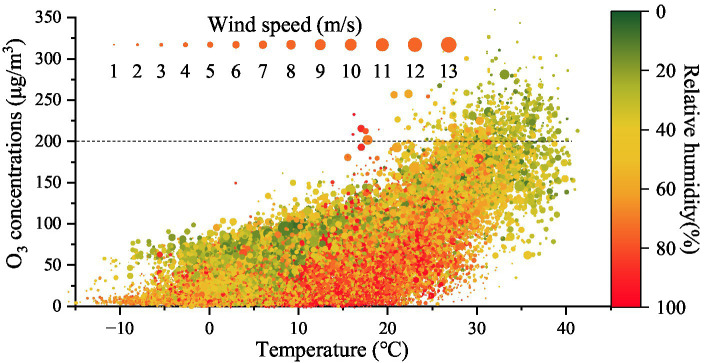
O_3_ concentration distribution under different temperatures, relative humidities, and wind speeds on the Fenwei Plain in 2017.

The O_3_ concentration distributions under different temperatures, relative humidities, and wind speeds on the Fenwei Plain in 2017 are shown in Figure 9. The meteorological conditions for the formation of high O_3_ concentration in the Fenwei Plain are as follows: temperature ≥ 25°C, relative humidity 20–40%, and wind speed ≤5 m·s^−1^. Under the same relative humidity conditions, high O_3_ pollution mainly appeared in the high-temperature region.

### 3.3. Population health impact assessment

**Figure 10:**
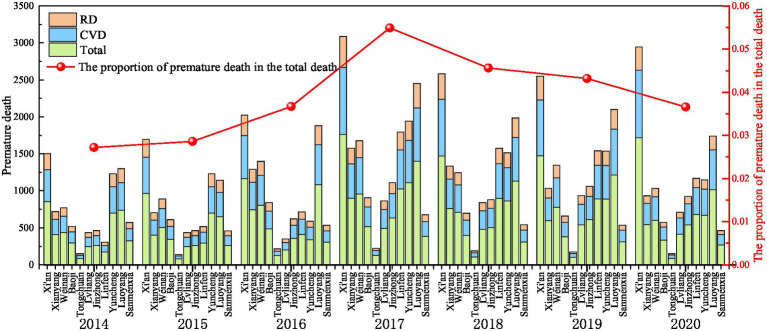
Premature deaths caused by O_3_ exposure on the Fen-Wei Plain from 2014 to 2020.

The number of premature deaths caused by O_3_ exposure fluctuated and increased on the Fen-Wei Plain from 2014 to 2020, which for each year were 7,955 (95% CI: 3,826–12,446), 8,279 (95% CI: 4150–12,951), 10,446 (95% CI: 5242–16,270), 16,300 (95% CI:8179–25,315), 13,372 (95% CI: 6689–20,810), 13,460 (95% CI: 6735–20,893), and 11,777 (95% CI: 5,879–18,235), accounting for 2.72, 2.86, 3.67, 5.49, 4.56, 4.32, and 3.66% of the total deaths in the region, respectively (Figure 10). Compared with 2014, the total number of premature deaths related to O_3_ exposure increased by 48.05% in 2020 in the Fenwei Plain region. In Linfen City, the increasing rate of premature deaths related to O_3_ exposure reached 283.39% (from 305 in 2014 to 1,168 in 2020) due to a large increase in O_3_ concentration. The proportion of total deaths in Linfen increased from 1.21 to 4.97%.

In 2014, the numbers of total, cardiovascular and respiratory deaths attributed to O_3_ pollution on the Fenwei Plain were 4,523 (95% CI: 2,458–6,571), 2,287 (95% CI: 851–3,706) and 1,145 (95% CI: 678–2,168), respectively. The largest number of excess deaths from three kinds of O_3_ pollution-related diseases was in Xi‘an, with 854, 432, and 217 people, respectively, while the least number of deaths was in Tongchuan. In 2020, the numbers of total, cardiovascular and respiratory deaths attributable to O_3_ pollution on the Fenwei Plain were 6,867 (95% CI: 3,739–9,965), 3,652 (95% CI: 1,363–5,905), and 1,257 (95% CI: 747–2,365), respectively, among which the number of deaths from respiratory diseases was basically the same as that in 2014. The excess deaths from total diseases, cardiovascular diseases, and respiratory diseases attributable to O_3_ pollution-related deaths in the 11 cities in 2020 showed the same trend as that in 2014. Fann et al. (47) estimated that approximately 4,700 excess deaths in the United States in 2005 were related to O_3_ pollution, based on model simulation and epidemiological assessment. Lelieveld et al. (48) estimated that approximately 2,395 excess deaths were related to O_3_ pollution in Beijing in 2013. However, the current O_3_ pollution is very serious and has become the primary pollutant harmful to human health. Since O_3_ is colorless and odorless in daily life, people tend to ignore its presence. In the future, collaborative control of PM_2.5_ and O_3_ pollution should be strengthened to ensure residents’ lives and health.

**Figure 11:**
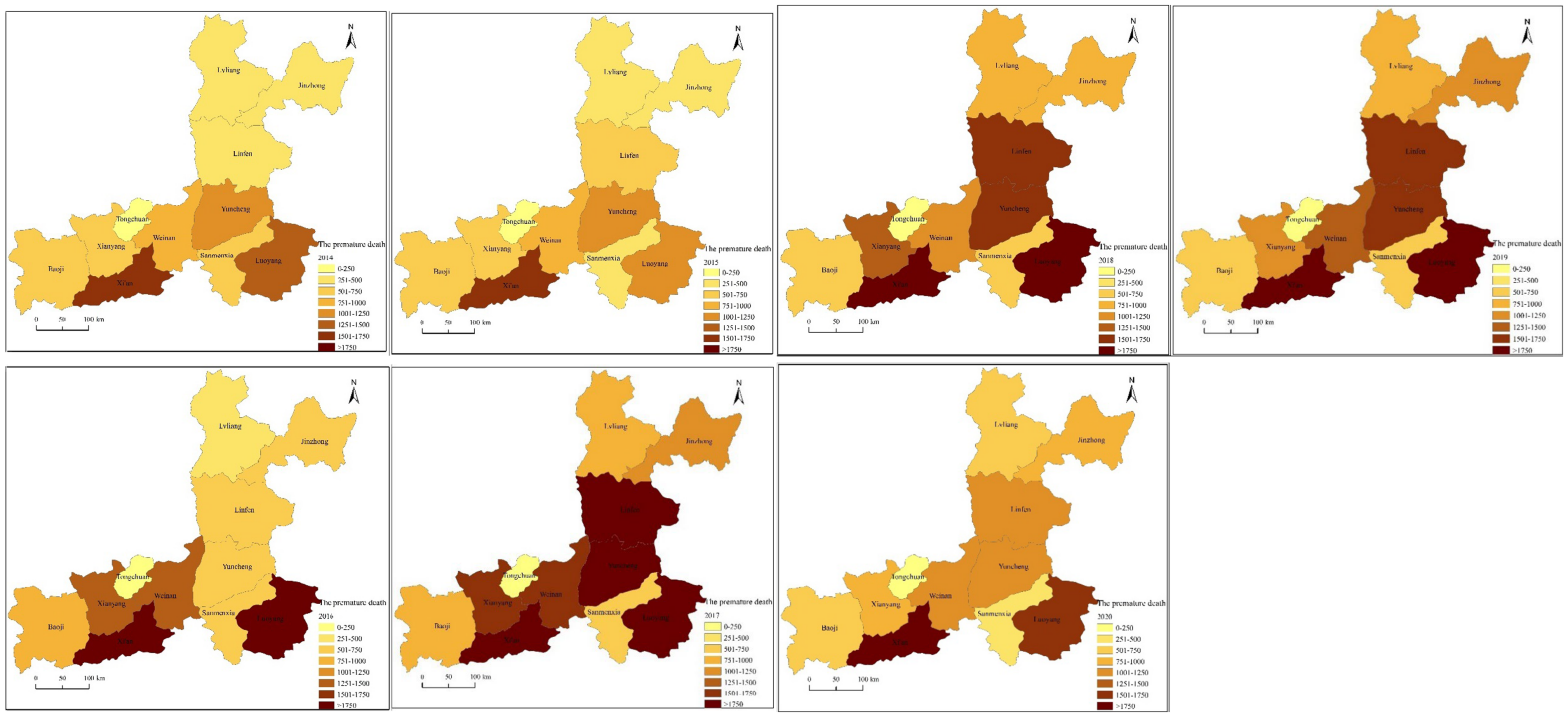
Spatial distribution of premature deaths attributed to O_3_ exposure in cities on the Fenwei Plain from 2014 to 2020.

The spatial distribution of premature deaths attributed to O_3_ exposure in cities on the Fenwei Plain from 2014 to 2020 is shown in Figure 11. After relatively light O3 pollution in 2014, the number of premature deaths related to O_3_ increased significantly, reaching the highest value during the study period in 2017, which were 3,085, 1,576, 1,675, 904, 221, 863, 1,111, 1,795, 1,941, 2,449, and 674 in the 11 cities, respectively. Compared with the lowest value, it increased by 105.29, 119.72, 118.00, 73.96, 49.19, 98.93, 141.56, 489.51, 57.84, 88.72, and 18.22%, respectively. Xi’an City has a large population and significant population growth during the study period, and the number of premature deaths attributed to O_3_ concentrations exceeding the standard value was the largest (number of premature deaths in 2020 increased by 1,443 compared with 2014). With the aggravation of O_3_ pollution and the gradual expansion of the pollution range, the numerical and spatial range of early death caused by O_3_ exposure in other cities on the Fenwei Plain also increased each year. Especially in the cities of Linfen, Yuncheng, Jinzhong, and Lvliang in the central and southern parts of Shanxi Province, the increase in the number of premature deaths was obviously related to severe O_3_ pollution and its increasing concentration. Linfen saw the largest increase in the number of premature deaths attributed to O_3_ exposure, with an increase of 863, and the largest increase in proportion (nearly 300%). This was followed by Jinzhong and Lvliang, where the number of premature deaths attributed to O_3_ exposure increased by 100.73 and 63.01%, respectively, during 2014–2020.

### 3.4. Health and economic loss assessment

**Figure 12:**
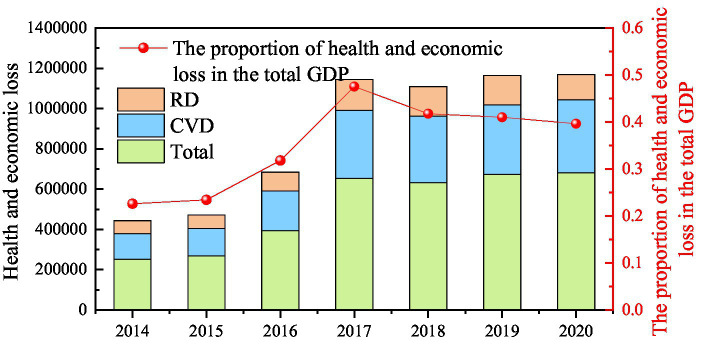
Health and economic losses caused by exposure to O_3_ during 2014–2020.

The health and economic losses caused by exposure to O_3_ during 2014–2020 are shown in Figure 12. The health and economic losses attributed to O_3_ air pollution on the Fenwei Plain during the study period were 44.22 (95% CI: 22.17–69.18), 47.16 (95% CI: 23.64–73.77), 68.28 (95% CI: 34.27–106.31), 114.44 (95% CI: 57.42–177.76), 110.85 (95% CI: 55.45–172.52), 116.41 (95% CI: 58.24–180.74), and 116.81 (95% CI: 58.00–180.88) billion yuan in each year, respectively, accounting for 0.22% (95% CI: 0.11–0.35%), 0.23% (95% CI: 0.12–0.37%), 0.32% (95% CI: 0.16–0.49%), 0.48% (95% CI: 0.24–0.74%), 0.42% (95% CI: 0.21–0.65%), 0.41% (95% CI: 0.21–0.64%), and 0.39% (95% CI: 0.20–0.61%) of the total GDP in each year, respectively. From 2014 to 2020, the economic loss due to poor health from O_3_ and the number of people at risk of poor health from O_3_ on the Fen-Wei Plain showed the same trend, and both showed an overall increasing trend. The main reason for this was that the O_3_ concentration showed an obvious increasing trend when the unit economic value of the permanent population and mortality fluctuated little.

From 2014 to 2020, the *per capita* health economic losses of cities on the Fenwei Plain due to O_3_ pollution showed an increasing and fluctuating trend, with an obvious rising trend year by year from 2014 to 2017, and then the rising trend slowed down (See Supplementary Table 1). However, the health loss in cities on the Fenwei Plain has still been large in recent years, among which Xi’an, Luoyang and Xianyang are still the cities with the largest economic loss on the Fenwei Plain. In 2020, the economic losses of the three cities were 3.768 billion yuan (95% CI: 18.71–58.36), 2.008 billion yuan (95% CI: 9.98–31.09), and 1.013 billion yuan (95% CI: 5.03–15.69), respectively, accounting for approximately 58.13% of the total health benefits. The health economic losses of Tongchuan and Sanmenxia were relatively low, accounting for approximately 5.32% of the total of the Fenwei Plain in 2020. Due to the differences in the number of exposed populations in each city, the ranking of health economic loss and *per capita* health loss of cities on the Fenwei Plain is also different, which is mainly caused by differences in permanent populations and O_3_ concentrations among different cities on the Fenwei Plain. For example, Yuncheng City ranked high in health economic loss, but its *per capita* health economic loss was relatively low.

In general, the trend of health economic loss attributable to O_3_ air pollution in cities on the Fenwei Plain from 2014 to 2020 was consistent with the overall trend of the Fenwei Plain, showing an overall rising trend. On the one hand, due to rapid social and economic development, the acceleration of urbanization, and the increase in the consumption of energy and fossil fuels, O_3_ pollution intensifies and increases the health risk of the exposed population, resulting in a large increase in the number of premature deaths. On the other hand, the growth of GDP, population and people’s WTP play an important role. In particular, with economic development, the implementation of the strategy of regional construction of megacenter cities will have a siphon effect on the population of surrounding cities. For example, the population of Xi’an increased from 8,657,200 in 2014 to 12,952,900 in 2020. This is also the main reason for the high number of premature deaths and economic health losses attributable to O_3_ pollution exposure in Xi’an.

### 3.5. Uncertainty analysis

This study mainly evaluated the excess cardiovascular disease deaths, respiratory disease deaths, and total deaths related to O_3_ pollution on the Fenwei Plain from 2014 to 2020. The results of this study are comparable with those of similar studies. However, due to the complexity of the health endpoint in the clinical population, they are difficult to verify through actual death population data. Therefore, the uncertainty factors in the evaluation process of this study were analyzed and examined.

The exposure response relationship of air pollutants is a key parameter to evaluate population health effects and an important source of uncertainty. Existing global studies have adopted consistent exposure response coefficients for different regions (48), and existing national scale assessments have also adopted foreign exposure response coefficients for air pollutants and disease endpoints (18, 23, 49). In view of the lack of studies on long-term O_3_ exposure and population mortality in China, the analysis results of Zeng et al. (14) were adopted in this study to evaluate the impact of O_3_ on population health. Subject to the types of diseases related to air pollutants contained in the epidemiological survey results, this study mainly analyzed the excess deaths of respiratory diseases, cardiovascular diseases, and total premature death. Therefore, future studies on the relationship between different diseases and exposure responses should be strengthened to clarify the relationship between O_3_ in China and exposure responses to population mortality to provide a basis for health risk assessment.

The accuracy of population and pollutant concentration distribution data is also an important factor in assessing uncertainty due to the large difference in the spatial distribution of exposed populations and air pollutants. The population data in this study came from the permanent population data of the 11 cities on the Fenwei Plain. The total population is in a dynamic changing process and has spatial mobility. This study did not consider the dynamic and time changes in the grid population and chose the regional total population for calculation, which has some errors. In addition, the O_3_ data in this study came from atmospheric environmental monitoring sites, and the lack of monitoring data in rural areas may underestimate the actual O_3_ concentration. Therefore, it is necessary to accurately predict the concentration of air pollutants by combining monitoring data and atmospheric chemical models in the future, improve the resolution of pollutant concentration and population data, and evaluate the health benefits of air pollution more accurately.

## 4. Conclusion

1. From 2014 to 2020, the average annual concentration of O_3__8h_max pollutants on the Fenwei Plain showed an overall upward trend, increasing by 32.39% in 2020 compared with 2014. The number of days exceeding the standard for O_3_ pollution was on the rise and fluctuated. In particular, the annual average number of days exceeding the standard for O_3_ pollution on the Fenwei Plain in 2017 reached 70.8, which increased by 33.7 days compared with 2016.
2. The monthly variation in the O_3__8h_max concentration on the Fenwei Plain presented an “M” bimodal pattern. The diurnal variation was significantly affected by near-surface atmospheric photochemical processes, showing a relatively obvious single-peak and single-valley distribution, and the O_3_ concentration was low in the morning and night. From the perspective of seasonal variations, the average mass concentration of ground-level O_3_ in different seasons was ranked as summer > spring > autumn > winter. In summer, temperature is high, radiation is strong, photochemical reactions are strong, and precursor pollutants easily form O_3_ under intense photochemical reactions.
3. The concentration of O_3__8h_max increased with increasing sunshine duration and temperature, showing a significant positive correlation. With increasing relative humidity and wind speed, the average concentration of O_3__8h_max first increased and then decreased. When the temperature is higher than 25°C, the relative humidity range is 20–40%, and the wind speed is less than 5 ms^-1^, high concentrations of O_3_ pollution easily occur.
4. From 2014 to 2020, the number of premature deaths caused by O_3_ exposure on the Fenwei Plain fluctuated and increased, reaching 7,955 (95% CI: 3,827–12,446), 8,279 (95% CI: 4,150–12,951), 10,446 (95% CI: 5,242–16,270), 16,300 (95% CI: 8,179–25,315), 13,372 (95% CI: 6,689–20,810), 13,460 (95% CI: 6,735–20,893), and 11,777 (95% CI: 5,849–18,235), accounting for 2.72, 2.86, 3.67, 5.49, 4.56, 4.32, and 3.66% of total deaths in the region, respectively. Among them, due to the obvious increase in O_3_ concentration and population in Xi’an, the number of premature deaths attributed to O_3_ concentrations exceeding the standard value was the largest, accounting for 25.02% of the total deaths in the region in 2020. With the worsening of O_3_ pollution, the scope of pollution is gradually expanding. Especially in Linfen city, due to the rapid growth of O_3_ concentration, the number of premature deaths in 2020 increased by 283.39% compared with 2014.
5. On the Fenwei Plain, the health economic loss due to O_3_ pollution increased significantly in 2017 and then slowed down. The total health economic losses attributable to O_3_ air pollution during the study period were 44.22 (95% CI: 22.17–69.18), 47.16 (95% CI: 23.64–73.77), 68.28 (95% CI: 34.27–106.31), 114.44 (95% CI: 57.42–177.76), 110.85 (95% CI: 55.45–172.52), 116.41 (95% CI: 58.24–180.74), and 116.81 (95% CI: 58.00–180.88) billion yuan, respectively. Xi’an, Luoyang, and Xianyang are still the cities with the largest health and economic losses on the Fenwei Plain, accounting for 58.13% of the total health losses in 2020. More densely populated and economically developed cities suffer greater health risks and economic losses at the same level of exposure. Therefore, economically developed and densely populated cities should focus on air pollution prevention and control; promote the adjustment of urban industrial structure, energy structure and traffic structure; and do a better job in regard to pollutant reduction to reduce health risks and economic losses and achieve coordinated economic and environmental development.

## Data availability statement

Publicly available datasets were analyzed in this study. This data can be found at: http://www.cnemc.cn/.

## Author contributions

XS: conceptualization and data curation. XS and YH: methodology, investigation, resources, writing—original draft preparation, and writing—review and editing. YH: software. All authors contributed to the article and approved the submitted version.

## Funding

This research was funded by “the National Natural Science Foundation of China” (72104132), “Humanities and Social Science Fund of Ministry of Education of China” (21YJCZH136), and supported by “Fundamental Research Program of Shanxi Province” (20210302124201).

## Conflict of interest

The authors declare that the research was conducted in the absence of any commercial or financial relationships that could be construed as a potential conflict of interest.

## Publisher’s note

All claims expressed in this article are solely those of the authors and do not necessarily represent those of their affiliated organizations, or those of the publisher, the editors and the reviewers. Any product that may be evaluated in this article, or claim that may be made by its manufacturer, is not guaranteed or endorsed by the publisher.
