# Supplementary material for: An assessment of O3-related health risks and economic losses in typical regions of China
Source: Front Public Health. 2023 Sep 5;11:1194340. doi: 10.3389/fpubh.2023.1194340 (PMC10508848; doi:10.3389/fpubh.2023.1194340)
Supplement: Supplementary file 1 [file Table_1.DOCX]

Table Appendix 1 The health economic losses attributed to O_3_ pollution in cities on the Fenwei Plain from 2014 to 2020.

| 2014 | | | | | | 2015 | | | | |
| --- | --- | --- | --- | --- | --- | --- | --- | --- | --- | --- |
| Cities | Health economic losses (yuan) | Ranking | As a percentage of GDP | Per capita health economic losses (yuan) | Ranking | Health economic losses (yuan) | Ranking | As a percentage of GDP | Per capita health economic losses (yuan) | Ranking |
| Xi’an | 118784.3(50434.98,185862.9) | 1 | 0.213(0.09,0.333) | 79.0514 | 1 | 141307(60103.27,220880.1) | 1 | 0.238(0.101,0.372) | 83.23622 | 1 |
| Xianyang | 42152.86(17881.44,66028.62) | 4 | 0.232(0.099,0.365) | 58.7519 | 4 | 45982.99(19526.03,72017.77) | 3 | 0.239(0.101,0.374) | 65.14293 | 5 |
| Weinan | 32177.69(13649.47,50405.41) | 6 | 0.237(0.10,0.372) | 41.8643 | 8 | 37689.16(16016.01,58976.42) | 6 | 0.276(0.117,0.432) | 42.43062 | 8 |
| Baoji | 30189.16(12803.82,47299.78) | 7 | 0.205(0.087,0.322) | 58.0650 | 5 | 40034.25(17010.3,62655.89) | 5 | 0.253(0.107,0.395) | 65.88891 | 4 |
| Tongchuan | 7712.017(3274.659,12066.24) | 11 | 0.276(0.117,0.432) | 51.8426 | 6 | 6931.375(2945.488,10846.29) | 11 | 0.260(0.111,0.407) | 49.42719 | 6 |
| Lvliang | 15159.97(6431.744,23768.38) | 9 | 0.137(0.058,0.215) | 34.9125 | 10 | 11795.77(5004.452,18493.85) | 10 | 0.123(0.052,0.193) | 27.16498 | 11 |
| Jinzhong | 19678.11(8354.993,30824.08) | 8 | 0.188(0.080,0.296) | 42.7654 | 7 | 19572.95(8310.342,30659.35) | 8 | 0.187(0.079,0.293) | 42.53689 | 7 |
| Linfen | 11083.97(4690.75,17411.02) | 10 | 0.091(0.039,0.144) | 36.3972 | 9 | 17421.01(7391.624,27310.62) | 9 | 0.150(0.063,0.235) | 33.86067 | 9 |
| Yuncheng | 42907.08(18271.82,66974.57) | 3 | 0.36(0.152,0.557) | 34.8771 | 11 | 40626.09(17300.47,63414.12) | 4 | 0.346(0.147,0.540) | 33.02303 | 10 |
| Luoyang | 82281.23(34958.12,128650) | 2 | 0.25(0.106,0.391) | 63.3884 | 3 | 77832.68(33078.85,121776.7) | 2 | 0.224(0.095,0.351) | 68.14733 | 3 |
| Sanmenxia | 40070.72(17056.19,62514.78) | 5 | 0.32(0.138,0.504) | 70.2644 | 2 | 32399.71(13784.61,50628.32) | 7 | 0.258(0.110,0.405) | 70.66524 | 2 |

| 2016 | | | | | | 2017 | | | | |
| --- | --- | --- | --- | --- | --- | --- | --- | --- | --- | --- |
| Cities | Health economic losses (yuan) | Ranking | As a percentage of GDP | Per capita health economic losses (yuan) | Ranking | Health economic losses (yuan) | Ranking | As a percentage of GDP | Per capita health economic losses (yuan) | Ranking |
| Xi’an | 179739.4(77158.15,279933.1) | 1 | 0.281(0.121,0.437) | 88.88 | 1 | 299102.2(128519.7,464729.7) | 1 | 0.403(0.173,0.626) | 96.95819 | 1 |
| Xianyang | 92784.24(39869.32,144339.6) | 3 | 0.458(0.197,0.713) | 71.87186 | 5 | 116980.6(50326.33,181499.5) | 3 | 0.610(0.263,0.947) | 74.20549 | 5 |
| Weinan | 63305.53(27203.45,98476.37) | 5 | 0.449(0.193,0.698) | 45.30485 | 10 | 88864.72(38172.61,138120.8) | 4 | 0.574(0.247,0.893) | 53.03415 | 8 |
| Baoji | 63938.62(27441.82,99604.43) | 4 | 0.375(0.161,0.585) | 76.04712 | 4 | 84523.66(36227.22,131715.9) | 5 | 0.441(0.189,0.688) | 93.45094 | 2 |
| Tongchuan | 11179.68(4802.917,17395.8) | 10 | 0.414(0.178,0.643) | 52.22787 | 6 | 14049.85(6026.856,21872.92) | 11 | 0.467(0.200,0.727) | 63.3048 | 6 |
| Lvliang | 10065.35(4301.382,15759.86) | 11 | 0.101(0.043,0.158) | 28.84524 | 11 | 38379.2(16441.15,59843.19) | 10 | 0.293(0.125,0.457) | 44.42845 | 9 |
| Jinzhong | 28048.55(12023.38,43757.59) | 7 | 0.257(0.110,0.401) | 45.23815 | 7 | 65125.15(27991.52,101153.4) | 8 | 0.507(0.218,0.787) | 58.59114 | 7 |
| Linfen | 25029.95(10720.62,39086.13) | 8 | 0.208(0.089,0.324) | 35.08772 | 9 | 72363.54(31170.65,112111) | 7 | 0.548(0.236,0.849) | 40.30842 | 10 |
| Yuncheng | 20693.51(8849.092,32375.67) | 9 | 0.169(0.072,0.265) | 35.2064 | 8 | 77565.61(33373.38,120329.8) | 6 | 0.580(0.249,0.900) | 39.94341 | 11 |
| Luoyang | 147009.4(63205.95,228541.6) | 2 | 0.384(0.165,0.598) | 78.24674 | 2 | 227905.2(98044.13,353616.3) | 2 | 0.531(0.229,0.824) | 93.03307 | 3 |
| Sanmenxia | 41014.77(17610.33,63862.66) | 6 | 0.309(0.133,0.482) | 77.15718 | 3 | 59523.2(25557.44,92563.5) | 9 | 0.411(0.177,0.639) | 88.28226 | 4 |

| 2018 | | | | | | 2019 | | | | |
| --- | --- | --- | --- | --- | --- | --- | --- | --- | --- | --- |
| Cities | Health economic losses (yuan) | Ranking | As a percentage of GDP | Per capita health economic losses (yuan) | Ranking | Health economic losses (yuan) | Ranking | As a percentage of GDP | Per capita health economic losses (yuan) | Ranking |
| Xi’an | 290165.4(124633.2,451772.3) | 1 | 0.34(0.15,0.53) | 112.4231 | 2 | 315916.3(136802.9,490766.8) | 1 | 0.34(0.15,0.52) | 123.91 | 1 |
| Xianyang | 132871(57153.24,206527) | 3 | 0.62(0.27,0.97) | 99.59842 | 4 | 110510.1(47835.5,171754.8) | 3 | 0.49(0.22,0.78) | 106.9684 | 3 |
| Weinan | 81691.04(35062.55,127298) | 4 | 0.47(0.20,0.74) | 65.63876 | 8 | 102710.9(44484.6,159528.4) | 4 | 0.56(0.24,0.87) | 76.32746 | 6 |
| Baoji | 78478.8(33634.34,122503.6) | 5 | 0.37(0.16,0.58) | 112.7346 | 1 | 82313.93(35560.45,128226.9) | 5 | 0.37(0.16,0.58) | 125.1642 | 2 |
| Tongchuan | 14140.79(6068.864,22037.51) | 11 | 0.43(0.18,0.66) | 76.20501 | 6 | 16312(7057.757,25364.98) | 11 | 0.44(0.19,0.69) | 93.59623 | 5 |
| Lvliang | 42761.46(18344.03,66675.41) | 10 | 0.30(0.13,0.47) | 50.90056 | 9 | 52636.14(22786.34,81797.88) | 9 | 0.35(0.15,0.54) | 56.31863 | 9 |
| Jinzhong | 61812.48(26551.7,96231.43) | 8 | 0.43(0.18,0.66) | 70.25964 | 7 | 74326.99(32249.95,115199.5) | 7 | 0.51(0.22,0.79) | 70.25012 | 8 |
| Linfen | 73240.54(31545.79,113664.1) | 7 | 0.51(0.22,0.79) | 46.54754 | 11 | 72302.52(31400.78,111940.8) | 8 | 0.49(0.22,0.77) | 46.97867 | 11 |
| Yuncheng | 73287(31501.66,114006.2) | 6 | 0.49(0.21,0.76) | 48.42938 | 10 | 77771.91(33716.49,120655.8) | 6 | 0.50(0.22,0.78) | 50.63557 | 10 |
| Luoyang | 208101(89464.65,323663) | 2 | 0.45(0.19,0.70) | 104.964 | 3 | 218674.1(94853.71,339037) | 2 | 0.44(0.19,0.68) | 104.2669 | 4 |
| Sanmenxia | 51923.93(22289.51,80898.42) | 9 | 0.39(0.17,0.61) | 95.91298 | 5 | 40639.88(17590,63168.44) | 10 | 0.29(0.13,0.45) | 75.97391 | 7 |

| 2020 | | | | | |
| --- | --- | --- | --- | --- | --- |
| Cities | Health economic losses (yuan) | Ranking | As a percentage of GDP | Per capita health economic losses (yuan) | Ranking |
| Xi’an | 376795(165602,583631.3) | 1 | 0.38(0.17,0.58) | 127.8879 | 1 |
| Xianyang | 101332(44546.02,156914.3) | 3 | 0.46(0.20,0.71) | 108.8395 | 4 |
| Weinan | 83472.64(36677.74,129328.8) | 4 | 0.45(0.20,0.69) | 80.99654 | 7 |
| Baoji | 77995.52(34221.48,121045.1) | 5 | 0.34(0.15,0.53) | 136.189 | 2 |
| Tongchuan | 15474.53(6798.42,23979.87) | 11 | 0.41(0.18,0.63) | 103.2217 | 5 |
| Lvliang | 50757.43(22294.88,78673.23) | 9 | 0.33(0.14,0.51) | 71.70546 | 9 |
| Jinzhong | 66728.32(29368.5,103190.7) | 8 | 0.45(0.20,0.70) | 72.245 | 8 |
| Linfen | 70833.44(31194.72,109460.4) | 7 | 0.47(0.21,0.73) | 60.66926 | 11 |
| Yuncheng | 77205.36(33945.15,119532.3) | 6 | 0.47(0.21,0.73) | 67.31492 | 10 |
| Luoyang | 200836.7(88319.85,310873.2) | 2 | 0.39(0.17,0.61) | 115.4152 | 3 |
| Sanmenxia | 46635.36(20495.51,72238.44) | 10 | 0.32(0.14,0.49) | 101.323 | 6 |
